# Sonographic evaluation of intracranial hemodynamics and pressure after out-of-hospital cardiac arrest: An exploratory sub-study of the TAME trial

**DOI:** 10.1016/j.ccrj.2024.06.001

**Published:** 2024-06-27

**Authors:** Halvor Ø. Guldbrandsen, Peter Juhl-Olsen, Glenn M. Eastwood, Kasper L. Wethelund, Anders M. Grejs

**Affiliations:** a Department of Intensive Care Medicine, Aarhus University Hospital, Aarhus, Denmark; b Department of Clinical Medicine, Aarhus University, Aarhus, Denmark; c Department of Cardiothoracic- and Vascular Surgery, Anaesthesia Section, Aarhus University Hospital, Aarhus, Denmark; d Department of Intensive Care, Austin Hospital, Melbourne, Victoria, Australia; e Australian and New Zealand Intensive Care Research Centre, School of Public Health and Preventive Medicine, Monash University, Victoria, Australia

**Keywords:** Out-of-hospital cardiac arrest, Post-cardiac arrest syndrome, Hypercapnia, Ultrasonography, Doppler, transcranial, Intracranial pressure

## Abstract

**Objective:**

Targeted mild hypercapnia is a potential neuroprotective therapy after cardiac arrest. In this exploratory observational study, we aimed to explore the effects of targeted mild hypercapnia on cerebral microvascular resistance assessed by middle cerebral artery pulsatility index (MCA PI) and intracranial pressure estimated by optic nerve sheath diameter (ONSD) in resuscitated out-of-hospital cardiac arrest (OHCA) patients.

**Design, setting, participants and interventions:**

Comatose adults resuscitated from OHCA were randomly allocated to targeted mild hypercapnia (PaCO_2_ 50–55 mmHg) or targeted normocapnia (PaCO_2_ 35–45 mmHg) for 24 h in the TAME trial.

**Main outcome measures:**

Using transcranial Doppler and transorbital ultrasound, we obtained MCA PI and ONSD at 4, 24, and 48 h after randomization. Ultrasound parameters were compared between groups using a linear mixed effects model.

**Results:**

Twelve consecutive patients were included, with seven patients in the mild hypercapnia group. MCA PI decreased from 4 to 24 h (p = 0.019) and was lower over the first 24 h in patients allocated to targeted mild hypercapnia compared with targeted normocapnia (p = 0.047). ONSD did not differ between groups or over time.

**Conclusion:**

Cerebral microvascular resistance assessed by MCA PI decreased over 24 h and was lower in OHCA patients treated with targeted mild hypercapnia compared with targeted normocapnia. Targeted mild hypercapnia did not exert substantial effect on intracranial pressure as estimated by ONSD.

## 1 Introduction

Hypoxic ischemic brain injury (HIBI) is the leading cause of mortality, withdrawal of life-sustaining treatment, and long-term disability in resuscitated cardiac arrest patients.^1^^,^^2^ There is currently no single treatment available to directly reduce HIBI after cardiac arrest.^2^ One feature of HIBI is altered cerebral perfusion,characterized by an initial transient period of hyperemia immediately after return of spontaneous circulation (ROSC). Within a few hours, this is followed by a global cerebral hypoperfusion.^2^

The Targeted Therapeutic Mild Hypercapnia After Resuscitated Cardiac Arrest (TAME) trial compared targeted mild hypercapnia (MH) with targeted normocapnia (TN) in comatose adults resuscitated from out-of-hospital cardiac arrest (OHCA) patients and showed no difference in neurological outcome.^3^ A speculated neuroprotective effect of MH is counteraction of post-resuscitation cerebral hypoperfusion through dilatation of cerebral arterioles, resulting in lower microvascular resistance with a consequent increase of cerebral blood flow.^4^^,^^5^

The pulsatility index of the middle cerebral artery (MCA PI), obtained with ultrasonography, serves as a surrogate marker for downstream cerebral microvascular resistance.[6], [7], [8] Pulsatility index has a high correlation with vascular resistance across changes in resistance, regardless whether the changes are induced by alterations in perfusion pressure or flow.^9^^,^^10^ Increased microvascular resistance in disease states such as cerebral small vessel disease correlates with elevated MCA PI.^11^ Isolated hypercapnia was shown by transcranial Doppler to decrease MCA PI in small clinical studies and small experimental animal studies.^12^^,^^13^

Hypercapnia increases cerebral blood flow, cerebral blood volume and ultimately should also increase intracranial pressure (ICP).^4^^,^^14^ The ICP can be estimated non-invasively by measuring the optic nerve sheath diameter (ONSD) with transorbital ultrasonography.^15^^,^^16^

In normocapnic OHCA patients, elevated MCA PI and ONSD values are associated with poor neurological outcomes when measured within 24 h after ROSC.[17], [18], [19], [20]

Given the neutral findings of the TAME trial, we find it important to report on whether the speculated potentially beneficial effects on cerebral perfusion and potentially harmful effect on cerebral pressure were actually present.

This pre-planned sub-study aimed to evaluate the MCA PI and ONSD over time in comatose adults resuscitated after OHCA. We hypothesized that MCA PI decreases and ONSD increases more during the intervention period in patients allocated to targeted mild hypercapnia compared to target normocapnia in the TAME trial.^3^

## 2 Methods

### 2.1 Study design and participants

This was a single-center prospective observational sub-study of the phase III multi-center randomized controlled TAME trial (ClinicalTrials.gov Identifier: NCT03114033) conducted at Aarhus University Hospital, Denmark. The TAME trial protocol is described elsewhere.^3^ Briefly, comatose adults resuscitated after OHCA of a presumed cardiac or unknown cause with sustained ROSC (>20 min) were randomly assigned to MH (arterial partial pressure of carbon dioxide, PaCO_2_, target of 50–55 mmHg) or TN (PaCO_2_ target of 35–45 mmHg) for 24 h after randomization. This study was approved by the Central Denmark Region Committee on Health Research Ethics (identifier 1-10-72-199-18). Written informed consent was obtained from all subjects or next of kin. The study complies with the Helsinki II declaration. Reporting is consistent with the STROBE guidelines.

### 2.2 Ultrasound protocol

Transorbital and transcranial ultrasound scans were performed at 4, 24, and 48 h after randomization by a single sonographer (HG), who was blinded to treatment allocation. To evaluate the interrater reliability of measuring ONSD and MCA PI from obtained images, parameters were measured by two independent raters (KW and HG), who were both blinded to the other rater's measurements, PaCO_2_ management, and patient outcome. Transcranial and transorbital ultrasound data was analyzed offline using EchoPAC SW version 202 (GE Healthcare, Chicago, Illinois, USA).

### 2.3 Transcranial Doppler ultrasound

The M1 segment of the right MCA was examined through a temporal window of insonation using a 2.5–3.5 MHz phased array M5Sc transducer (GE Vivid S70N, GE Healthcare, Chicago, Illinois, USA). The vessel was located using color Doppler, and pulsed wave Doppler was used to quantify blood flow velocities. PI is defined according to the equation below and due to the indexing, the PI is independent of the insonation angle.

$$Pulsatility\;index=\frac{Peak\;systolic\;velocity-End\;diastolic\;velocity}{Mean\;velocity}$$

### 2.4 Transorbital ultrasound

A 7.5 MHz linear 11L transducer (GE Vivid S70N, GE Healthcare, Chicago, Illinois, USA) in B mode was oriented horizontally on the closed upper eyelid, pointing slightly inferior-medially to visualize the optic nerve sheath 3 mm posterior to the retina. Power was reduced to achieve a mechanical index <0.23 to minimize risk of damage to the globe according to guidelines.[21], [22], [23] Each eye was examined, and the ONSD value was averaged from both optical nerves.

### 2.5 Supplemental data collection and data storage

The following patient data was collected from the digital patient platform, MidtEPJ (Systematic A/S, Aarhus, Denmark): Age, sex, PaCO_2_ management, no-flow time, low-flow time, and survival at 30 days.

Further, PaCO_2_ and mean arterial blood pressure were collected at the time point of each ultrasound examination from the electronic patient data management system, PICIS PDM CareSuite (Harris Computer, Ottowa, Ontario, Canada).

All data was stored in REDCap version 11.1.29 (Vanderbilt University, Nashville, Tennessee, USA) according to local institutional guidelines.^24^^,^^25^

### 2.6 Endpoints

The primary endpoints were difference in MCA PI and ONSD during the first 24 h according to PaCO_2_ target groups.

### 2.7 Statistical analysis

Non-parametric data is presented as medians with bias-corrected and accelerated bootstrap 95% confidence intervals of medians or difference in medians using 10000 replicates unless otherwise stated. A Mann–Whitney-*U* test was used for testing of the null hypothesis in unpaired data, and Wilcoxon paired signed-rank test for paired data.

To test differences in MCA PI and ONSD between PaCO_2_ management groups throughout the intervention period (the first 24 h only) and investigate the interaction between PaCO_2_ management and time, we performed a linear mixed effects model with random intercepts using time and PaCO_2_ management as fixed effects with an interaction term between the two and subjects as random effects. We used Satterthwaite's approximation of the degrees of freedom for t statistics. Bonferroni method was used for post hoc correction for multiple comparisons. No correction was used for slopes and interaction terms, as these were the primary endpoints.

Previous studies in stroke patients and in healthy volunteers have shown that age, diabetes mellitus, and atherosclerotic disease are independent predictors of MCA PI.^26^^,^^27^ Although patients were randomized to PaCO_2_ targets, we performed a secondary linear mixed effects model with adjustment for age, diabetes mellitus, and atherosclerotic disease as fixed effects covariates to see if these parameters impacted the effects of PaCO_2_ target on MCA PI by changing the PaCO_2_ target coefficient estimate compared with the unadjusted model. Atherosclerotic disease was defined as the presence of ischemic heart disease and/or peripheral arterial disease and/or previous ischemic stroke. Because patients are randomized to PaCO_2_ targets independently of baseline characteristics, the endpoints were defined as the unadjusted model coefficients.

To evaluate interrater bias and variability, we constructed a Bland-Altman plot and line of agreement between the two raters. Mean interrater bias is reported with 95% limits of agreement (LOA). Interrater agreement was evaluated by using intra-class correlation (ICC) using a two-way random-effects single-rater type model for absolute agreement. ICC is presented with a 95% confidence interval.

Data analysis was performed in R Version 4.1.1 (R Foundation for Statistical Computing, Vienna, Austria). A p-value lower than 0.05 was considered significant. Data was analyzed based on the intention to treat.

As this was a pre-planned sub-study, no sample size calculation was performed, and all results are considered exploratory.

## 3 Results

### 3.1 Patient characteristics and assessment

Thirteen consecutive patients were screened and enrolled in the TAME trial from June through August 2021 at Aarhus University Hospital. Twelve of these patients were included in this exploratory sub-study with one patient excluded due to the unavailability of an experienced neurosonographer (Table 1). Seven patients were randomly allocated to the mild hypercapnia group and five patients to the normocapnia group. Six patients were alive at 30 days, of which there were three patients in each group.

**Table 1:** Patient characteristics.

	All patients	Targeted mild hypercapnia	Targeted normocapnia	P value
**Baseline characteristics**				
Number of patients	12	7	5	
Age (years)	71 (60–76)	70 (45–71)	75 (60–78)	
Female	1 (8%)	0 (0%)	1 (20%)	
Time to ROSC (min)	15 (7–26)	14 (3–15)	17 (6–35)	
Initial cardiac rhythm				
Shockable	9 (75%)	5 (71%)	4 (80%)	
Non-shockable	3 (25%)	2 (29%)	1 (20%)	
Cause of cardiac arrest				
Acute myocardial infarction	8 (67%)	5 (71%)	3 (60%)	
Hereditary cardiomyopathy	1 (8%)	1 (14%)	0 (0%)	
Complete heart block	1 (8%)	0 (0%)	1 (20%)	
Unknown etiology	2 (17%)	1 (14%)	1 (20%)	
Diabetes mellitus	2 (17%)	1 (14%)	1 (20%)	
Arterial hypertension	6 (50%)	2 (29%)	4 (80%)	
Hypercholesterolemia	6 (50%)	2 (29%)	4 (80%)	
Smoking status				
Never	2 (17%)	0 (0%)	2 (40%)	
Previous	6 (50%)	4 (57%)	2 (40%)	
Current	4 (33%)	3 (43%)	1 (20%)	
Peripheral arterial disease	2 (17%)	0 (0%)	2 (40%)	
Ischemic heart disease	8 (67%)	4 (57%)	4 (80%)	
Previous ischemic stroke	3 (25%)	1 (14%)	2 (40%)	
Chronic kidney disease	2 (17%)	1 (14%)	1 (20%)	
**Patient characteristics at each scan**				
PaCO_2_ (mmHg)				
4 h	47.2 (39.8–48.4)	48.5 (46.7–54.8)	39.8 (35.2–43.0)	0.010
24 h	42.9 (36.8–49.2)	49.2 (36.8–50.3)	37.5 (35.2–38.0)	0.030
48 h	40.6 (36.1–44.6)	44.6 (38.3–45.3)	37.1 (34.7–40.4)	0.06
MAP (mmHg)				
4 h	70.5 (65–81)	81 (60–82)	66 (60–70)	0.10
24 h	69 (62–71)	71 (44–74)	68 (61–69)	0.25
48 h	78 (65–86)	78 (65–78)	75 (53–88)	0.54
PaCO_2_ within target range				
4 h	5 (42%)	1 (14%)	4 (80%)	0.023
24 h	8 (67%)	3 (43%)	5 (100%)	0.08

MAP: mean arterial blood pressure. PaCO_2_: partial pressure of carbon dioxide.

Values are presented as median (95% confidence interval) or total number (%).

Transcranial and transorbital ultrasound examinations were performed in all patients at 4-h and 24-h time points. All patients had acceptable windows for transcranial Doppler. At the 48-h time-point, ultrasound examinations were performed in nine (75%) patients (one lost due to withdrawal of life-sustaining therapy, and two lost due to discharge from the intensive care unit). At 48 h, ONSD measurement could not be performed in two patients in which it was attempted due to head movement while weaning from sedation.

Although PaCO_2_ was higher in the MH group compared with the TN group at 4 and 24 h, only 1/7 and 3/7 in the MH group were within PaCO_2_ target range at 4 and 24 h, respectively (Table 1).

**Fig. 1:**
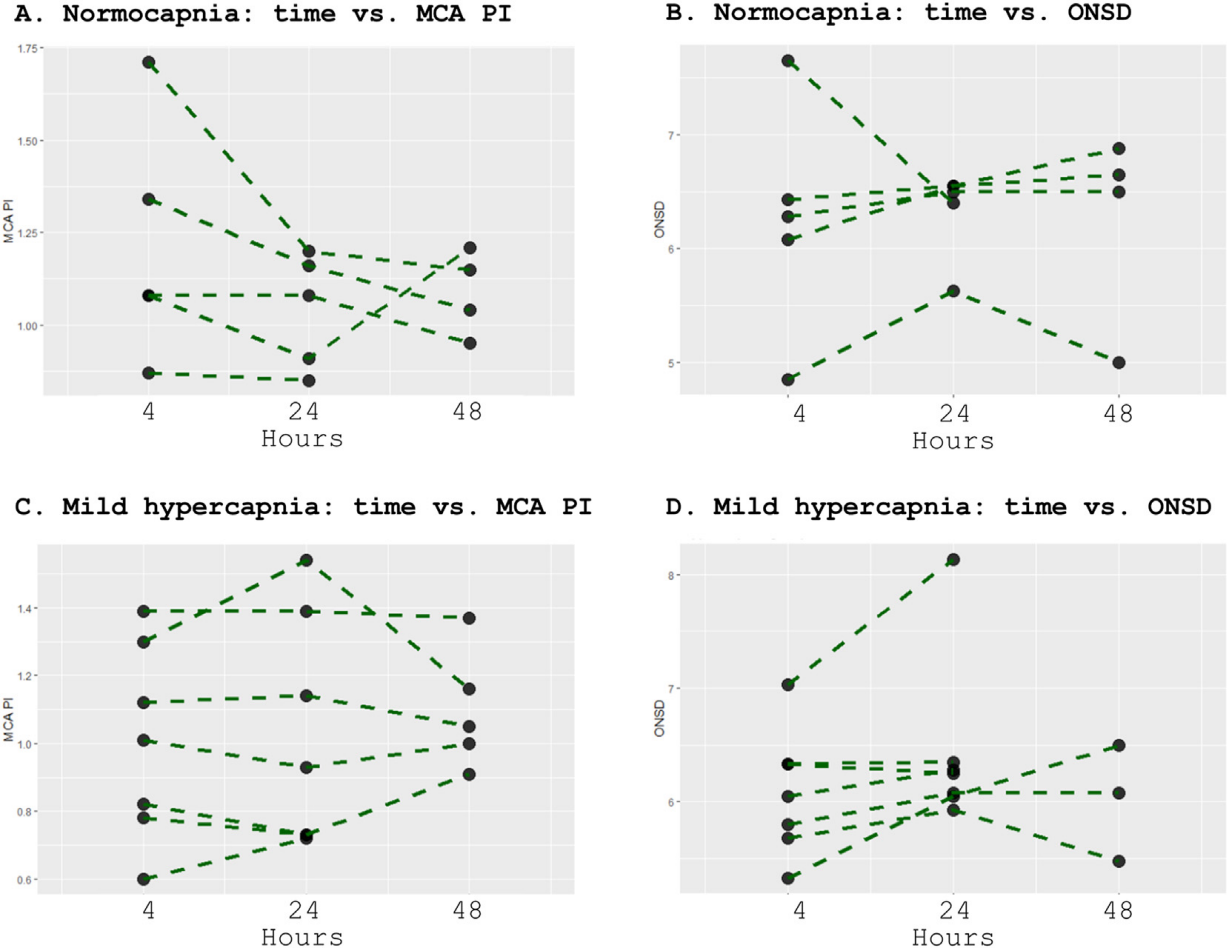
Title: Middle cerebral artery pulsatility index and optic nerve sheath diameter over time across PaCO_2_ management groups. Legend: MCA PI over time in the TN group (A) and MH group (C). ONSD over time in the TN group (B) and MH group (D). TN: targeted normocapnia. MH: targeted mild hypercapnia. MCA PI: middle cerebral artery pulsatility index. ONSD: optic nerve sheath diameter.

**Fig. 2:**
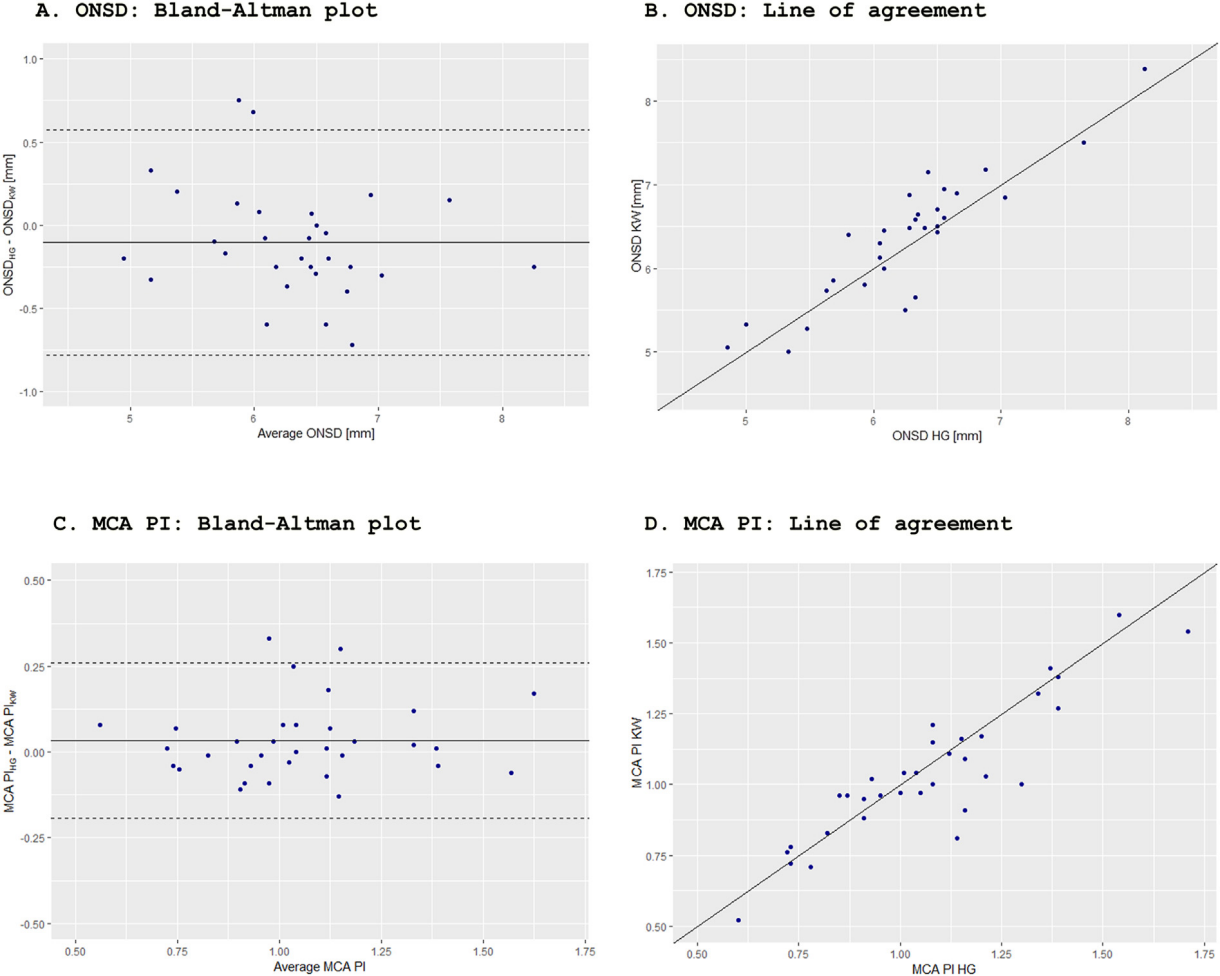
Title: Bland-Altman plots and lines of agreement between two raters of middle cerebral artery pulsatility index and optic nerve sheath diameter. Legend: Bland-Altman plots (A and C) of agreement in measured optic nerve sheath diameter (ONSD) (A) and middle cerebral artery pulsatility index (MCA PI) (C) between raters KW and HG. Dashed lines represent 95% limits of agreement (LOA). Limits of agreement (B and D) between measured values from the two raters (KW and HG). Interrater mean bias was −0.10 (95% LOA: −0.78; 0.57) mm for ONSD and 0.03 (95% LOA: −0.19; 0.26) for MCA PI. MCA PI: middle cerebral artery pulsatility index. ONSD: optic nerve sheath diameter.

**Fig. 3:**
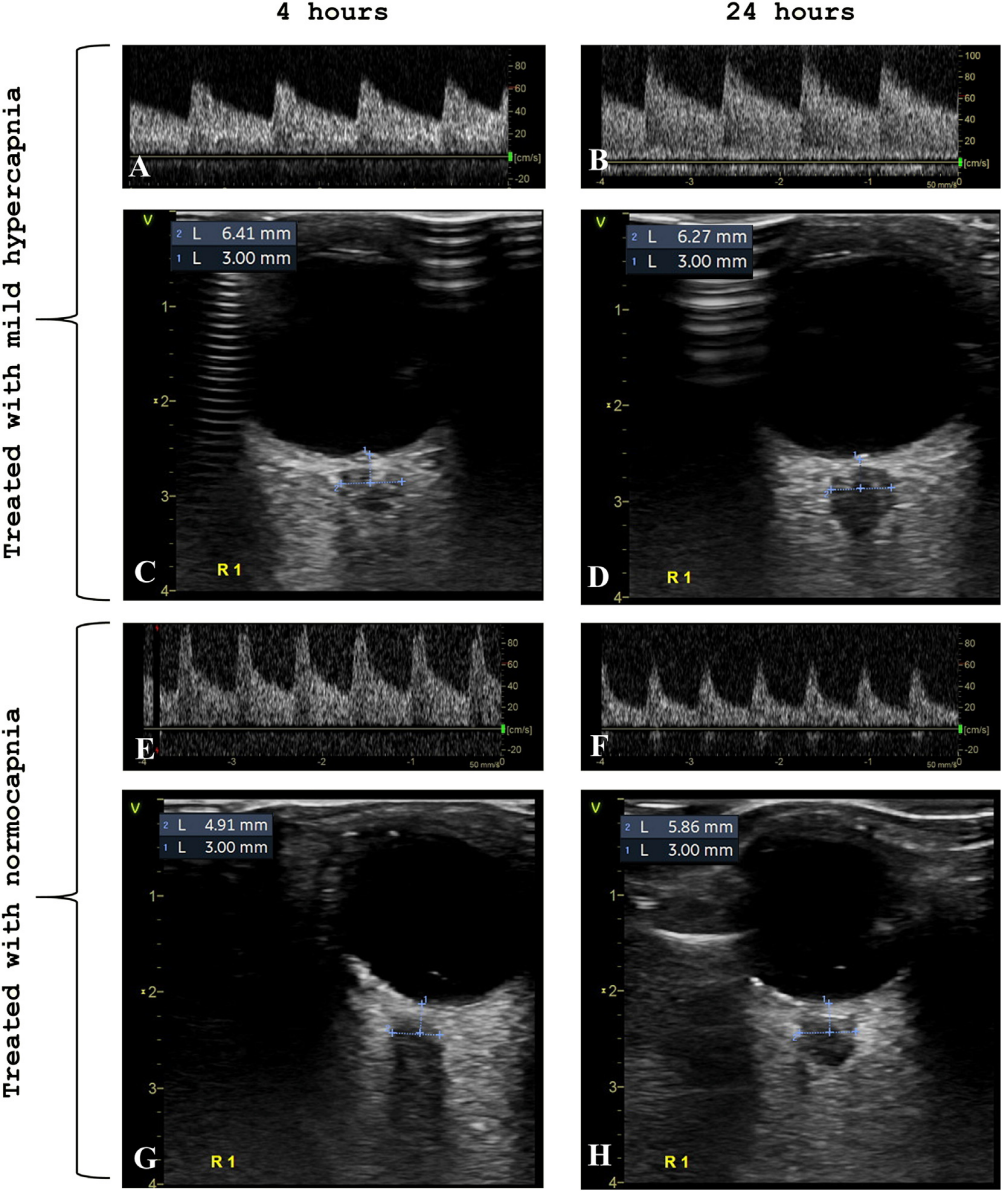
Title: Representative images of transcranial Doppler and transorbital ultrasound at 4 and 24 h for at surviving and a non-surviving patient. Legend: Representative ultrasound images for a survivor (A–D) and a non-survivor (E–H). For the survivor, MCA PI (A and B) and ONSD (C and D) decreased from 4 to 24 h. For the non-survivor, MCA PI (E and F) and ONSD (G and H) increase from 4 to 24 h. MCA PI is defined as the difference between peak systolic velocity and end diastolic velocity divided by mean velocity in a cardiac cycle. The indexing makes the parameter independent of the angle of insonation. MCA PI: middle cerebral artery pulsatility index. ONSD: optic nerve sheath diameter.

Fig. 1, Fig. 2 show ultrasound parameters over time and interrater variability, respectively. Fig. 3 shows representative images. Measurements for each individual patient are displayed in Table 4.

### 3.2 Middle cerebral artery pulsatility index

A linear mixed effects model showed that MCA PI was lower over the first 24 h in the MH group compared with TN (p = 0.047), as shown in Table 3. MCA PI decreases from 4 to 24 h (p = 0.019). MCA PI decreased less between 4 and 24 h in the MH group compared with the TN group (p = 0.037). A post-hoc subgroup analysis showed no difference in MCA PI between PaCO_2_ target groups at any time point. Non-parametric tests at individual time points are presented in Table 2. MCA PI did not change from 4 or 24 h–48 h in either group.

**Table 2:** Ultrasound measurements and outcomes.

	All patients	Targeted mild hypercapnia	Targeted normocapnia	P value
**Ultrasound measurements**				
Middle cerebral artery pulsatility index				
4 h	1.08 (0.82–1.21)	1.01 (0.60–1.12	1.08 (0.87–1.08)	0.33
24 h	1.01 (0.73–1.16)	0.93 (0.72–1.14)	1.08 (0.85–1.16)	0.75
48 h	1.05 (0.95–1.16)	1.05 (0.91–1.16)	1.10 (0.95–1.18)	1.00
Optic nerve sheath diameter (mm)				
4 h	6.18 (5.68–6.36)	6.05 (5.33–6.33)	6.28 (4.85–6.43)	0.63
24 h	6.32 (6.05–6.50)	6.25 (5.93–6.35)	6.50 (5.63–6.55)	0.33
48 h	6.30 (4.90–6.30)	6.08 (5.48–6.50)	6.58 (5.00–6.76)	0.48
**Patient outcomes**				
Survival at 30 days	6 (50%)	3 (43%)	3 (60%)	0.56

Values are presented as median (95% confidence interval) or total number (%).

### 3.3 Optic nerve sheath diameter

A linear mixed effects model showed no difference in ONSD between 4 and 24 h (p = 0.782) and no difference between PaCO_2_ target groups (p = 0.427). ONSD change from 4 to 24 h was not different between groups (p = 0.371). There was no difference in ONSD between groups at any time point and ONSD did not change from 4 or 24 h–48 h in either group.

### 3.4 Interrater agreement

The mean systematic interrater bias on acquired images was 0.03 for MCA PI and 0.10 mm for ONSD (Fig. 2). 95% limits of agreement (95% LOA) were −0.19 to 0.26 for MCA PI and −0.78 to 0.57 for ONSD. ICC between raters was 0.89 (95% CI: 0.80–0.94) for MCA PI and 0.89 (95% CI: 0.77–0.95) for ONSD.

## 4 Discussion

We measured MCA PI and ONSD at 4, 24, and 48 h in adult resuscitated cardiac arrest patients randomly allocated to targeted mild hypercapnia or targeted normocapnia. MCA PI decreased from 4 to 24 h. The decrease was less extreme in the MH group. MCA PI was lower over the first 24 h in the MH group. ONSD did not change over time and there was no difference in ONSD between groups.

For the age and gender distribution of the patient population, the reference value of MCA PI in healthy individuals is usually below 1.00.^28^ In our study at all three time points, the total patient population had a median MCA PI value above 1.00. This finding of elevated MCA PI in resuscitated cardiac arrest patients is aligned with previous findings of other groups^20^^,^[29], [30], [31] and is consistent with elevated cerebral microvascular resistance. These previous studies, in post-cardiac arrest patients with targeted normocapnia, showed early MCA PI median values of 1.12–1.86 when measured within 12 h and 1.00–1.05 when measured at 24 h after admission. In our pooled population, we found MCA PI to be 1.08 and 1.01 at 4 and 24 h, respectively. We did not find that the targeting mild hypercapnia induced cerebral arteriolar dilatation significantly counteracted the microvascular impairment, which occurs in resuscitated cardiac arrest patients.

Age, diabetes mellitus, and atherosclerotic disease are independent predictors of MCA PI in stroke patients. We showed that adjusting for these as covariates in our linear mixed effects model did little change to the estimates of the effect of PaCO_2_ target and time on MCA PI (Table 3). Thus, the observed effects on MCA PI were unlikely to be confounded by the named baseline characteristics.

**Table 3:** Fixed effects coefficients of linear mixed effects models.

Linear mixed effects models coefficients			
Coefficient	Estimate	95% confidence interval	P value
**MCA PI according to hypercapnia and time**			
Hypercapnia	−0.41	−0.81; −0.01	0.047
Time	−0.18	−0.31; −0.04	0.019
Interaction of time on hypercapnia	0.19	0.02; 0.38	0.037
**ONSD according to hypercapnia and time**			
Hypercapnia	−0.47	−1.67; 0.72	0.43
Time	0.07	−0.44; 0.58	0.78
Interaction of time on hypercapnia	0.29	−0.38; 0.96	0.37
**MCA PI according to hypercapnia and time with adjustment for age, diabetes mellitus, and atherosclerotic disease as covariates**			
Hypercapnia	−0.39	−0.82; 0.04	0.08
Time	−0.17	−0.31; −0.04	0.019
Interaction of time on hypercapnia	0.20	0.02; 0.38	0.037

MCA PI: middle cerebral artery pulsatility index. ONSD: optic nerve sheath diameter.

**Table 4:** MCA PI and ONSD measurements for all 12 patients.

ID	PaCO_2_ group	MCA PI			ONSD			30-day survival
		4 h	24 h	48 h	4 h	24 h	48 h	
1	TN	1.08	0.91	1.21	6.08	6.55	6.65	Yes
2	MH	1.30	1.54	1.16	5.68	5.93	5.48	No
3	MH	0.82	0.73	Discharged	6.33	6.35	Discharged	Yes
4	TN	1.08	1.08	0.95	4.85	5.63	5.00	No
5	MH	1.01	0.93	1.00	6.05	6.28	Movement	Yes
6	TN	0.87	0.85	Discharged	7.65	6.40	Discharged	Yes
7	MH	0.78	0.73	0.91	6.33	6.25	Movement	Yes
8	MH	1.39	1.39	1.37	5.80	6.08	6.08	No
9	MH	1.12	1.14	1.05	5.33	6.05	6.50	No
10	MH	0.60	0.72	WLST	7.03	8.13	WLST	No
11	TN	1.71	1.20	1.15	6.28	6.50	6.50	Yes
12	TN	1.34	1.16	1.04	6.43	6.55	6.88	No

Individual measurements for all 12 patients. PaCO2: partial pressure of carbon dioxide. MCA PI: middle cerebral artery pulsatility index. ONSD: optic nerve sheath diameter. TN: targeted normocapnia. MH: targeted mild hypercapnia. WLST: withdrawal of life-sustained therapy.

Mean systematic interrater bias on acquired images was 0.03 (95% LOA: −0.19-0.26) for MCA PI and 0.10 (95% LOA: −0.78-0.57) mm for ONSD (Fig. 2). Previous studies in healthy subjects showed interrater mean bias of −0.005 (95% LOA: −0.18-0.17) for MCA PI and between 0.08 (95% LOA: −0.64-0.48) mm and 0.09 (−0.90-0.71) mm for ONSD.^32^^,^^33^ ICC between raters was 0.89 (95% CI: 0.80–0.94) for MCA PI and 0.89 (95% CI: 0.77–0.95) for ONSD. Generally, ICC values between 0.75 and 0.90 are classified as good reliability. Previous studies of interrater reliabilities have shown moderate to good reliability of MCA PI and good to excellent reliability of ONSD.[32], [33], [34], [35], [36]

Hypercapnia causes an immediate decrease in cerebral microvascular resistance which weans over hours.^4^ We also observed lower MCA PI in patients allocated to mild hypercapnia over the first 24 h as an indication of lower cerebral microvascular resistance. MCA PI decreased more in the TN group, suggesting that with TN, a trend toward normalization of cerebral microvascular resistance takes longer time than with MH.

Elevated MCA PI in the first 24 h after resuscitation from cardiac arrest is associated with poor outcome.^20^^,^^30^ Although we show that MCA PI is lower over the first 24 h with MH, the TAME trial showed no benefit on neurological outcome with MH. This may suggest that elevated microvascular resistance is an indicator of poor prognosis due to early brain damage rather than a part of mechanism of late brain damage. In that case, elevated cerebral microvascular resistance is unlikely to be a potential target to inhibit neuroprotection.

It has previously been shown in normocapnic cardiac arrest patients that invasively measured ICP values peak between 24 and 48 h after resuscitation due to cerebral edema.[37], [38], [39] We did not see a difference in ONSD over the first 24 h following randomization. This might suggest that ONSD was not adequately sensitive to display the subtle delayed ICP rise. In the TAME trial, there were no reports of suspected or confirmed raised intracranial hypertension necessitating normocapnia listed as serious adverse events.

Lower MCA PI with MH and no difference in ONSD between groups during the intervention period may suggest that MH provides improved cerebral circulation without the cost of elevated ONSD.

The influence of MH on MCA PI and ONSD in healthy subjects is well established. Resuscitation from OHCA alters cerebral blood flow and intracranial pressure.^2^^,^^38^ The novelty of this study was the investigation of the effect of MH on MCA PI and ONSD after OHCA.

Our study findings suggest that MH did not exert a substantial effect on ICP as estimated by ONSD. Our findings also suggest that MH decreases cerebral microvascular resistance as evaluated by MCA PI. Furthermore, our findings suggest that MCA PI is elevated above normal reference values during the first hours after resuscitation, but trends toward normalization after 24 h.

### 4.1 Strengths and limitations

Our study has several strengths that include the use of serial measurements at fixed time points to follow dynamic changes in ultrasound parameters; the application of techniques like Transcranial Doppler and transorbital ultrasound, which are easily reproducible and carry no substantial interrater variability, and the fact that investigators obtained and assessed ultrasound parameters while blinded to PaCO_2_ target allocation and outcome.

We acknowledge several limitations. Mainly with 12 patients, we may have had compromised the ability to detect subtle intracranial hemodynamic and pressure effects occurring as a result of targeting either MH or NC. Results at 48 h should be interpreted with caution, as there was loss to follow-up. Although PaCO_2_ values were higher in the MH group at both time points, only 17% and 50% of the MH-treated patients were within PaCO_2_ target range at 4 h and 24 h respectively, which may have contributed to the observed similarity in ONSD between the groups (Table 5). The PaCO_2_ values measured in this sub-study were similar to those found in the main TAME trial. As this was analyzed using an intention-to-treat protocol rather than comparing patients based on their actual PaCO_2_ levels, our results may not be fully representative of the actual biological effect of mild hypercapnia on ONSD and MCA PI in adult resuscitated cardiac arrest patients. Although MCA PI was lower during the intervention period with MH compared with TN, there was no difference in MCA PI at any single time point. The exact time course of MCA PI during the intervention remains to be addressed with higher temporal resolution.

**Table 5:** MCA PI and ONSD stratified by whether the patient was within PaCO_2_ target range at the given time or not.

Middle cerebral artery pulsatility index		
	Within target PaCO_2_ range	Not within target PaCO_2_ range
Targeted normocapnia		
4 h	1.21 (0.87–1.52)	1.08 (n = 1)
24 h	All patients were within target PaCO_2_ range	
Targeted mild hypercapnia		
4 h	0.82 (n = 1)	1.06 (0.69–1.30)
24 h	1.14 (0.73–1.14)	0.83 (0.72–1.24)
**Optic nerve sheath diameter**		
Targeted normocapnia		
4 h	6.36 (6.08–7.65)	4.85 (n = 1)
24 h	All patients were within target PaCO_2_ range	
Targeted mild hypercapnia		
4 h	6.33 (n = 1)	5.92 (5.50–6.42)
24 h	6.08 (6.05–6.35)	6.26 (5.93–8.13)

PaCO2: partial pressure of carbon dioxide. Values are presented as median (95% confidence interval) unless stated otherwise.

## 5 Conclusion

In this pre-planned exploratory sub-study of the TAME trial, we showed that MCA PI was lower over the first 24 h in patients allocated to MH compared to targeted normocapnia, implying vasodilatation. Moreover, MCA PI decreases from 4 to 24 h after OHCA resuscitation and less so in patients allocated to MH compared with TN. We observed no difference in ONSD over time or between groups. These findings provide novel physiological data on the effect of targeting mild hypercapnia on MCA PI and ONSD, and due to the exploratory nature of the current study, it suggests the need for larger studies of these two investigational techniques.

## Ethical approval

As described in the methods section, this manuscript adheres to ethical guidelines and has ethical approval by the Central Denmark Region Committee on Health Research Ethics (identifier 1-10-72-199-18). Written informed consent was obtained from all subjects or next of kin. Reporting is consistent with the STROBE guidelines, the reporting checklist for observational studies.

## CRediT authorship contribution statement

Halvor Guldbrandsen: study conception and design; data collection; analysis and interpretation of results; draft manuscript preparation.

Peter Juhl-Olsen: analysis and interpretation of results; critical revision.

Glenn Eastwood: interpretation of results; critical revision.

Kasper Wethelund: data collection; analysis and interpretation of results.

Anders Grejs: study conception; analysis and interpretation of results; draft manuscript preparation.

All authors have made substantial contributions to the manuscript.

## Conflict of interest

The authors declare that they have no known competing financial interests or personal relationships that could have appeared to influence the work reported in this paper.
